# Are infant mortality rate declines exponential? The general pattern of 20^th ^century infant mortality rate decline

**DOI:** 10.1186/1478-7954-7-13

**Published:** 2009-08-23

**Authors:** David Bishai, Marjorie Opuni

**Affiliations:** 1 Department of Population, Family and Reproductive Health, Johns Hopkins University Bloomberg School of Public Health, 615 N. Wolfe Street, Baltimore, MD 21205, USA

## Abstract

**Background:**

Time trends in infant mortality for the 20^th ^century show a curvilinear pattern that most demographers have assumed to be approximately exponential. Virtually all cross-country comparisons and time series analyses of infant mortality have studied the logarithm of infant mortality to account for the curvilinear time trend. However, there is no evidence that the log transform is the best fit for infant mortality time trends.

**Methods:**

We use maximum likelihood methods to determine the best transformation to fit time trends in infant mortality reduction in the 20^th ^century and to assess the importance of the proper transformation in identifying the relationship between infant mortality and gross domestic product (GDP) per capita. We apply the Box Cox transform to infant mortality rate (IMR) time series from 18 countries to identify the best fitting value of lambda for each country and for the pooled sample. For each country, we test the value of *λ *against the null that *λ *= 0 (logarithmic model) and against the null that *λ *= 1 (linear model). We then demonstrate the importance of selecting the proper transformation by comparing regressions of ln(IMR) on same year GDP per capita against Box Cox transformed models.

**Results:**

Based on chi-squared test statistics, infant mortality decline is best described as an exponential decline only for the United States. For the remaining 17 countries we study, IMR decline is neither best modelled as logarithmic nor as a linear process. Imposing a logarithmic transform on IMR can lead to bias in fitting the relationship between IMR and GDP per capita.

**Conclusion:**

The assumption that IMR declines are exponential is enshrined in the Preston curve and in nearly all cross-country as well as time series analyses of IMR data since Preston's 1975 paper, but this assumption is seldom correct. Statistical analyses of IMR trends should assess the robustness of findings to transformations other than the log transform.

## Background

The assumption that infant mortality rate (IMR) declines are non-linear as an economy develops is enshrined in the Preston curve [1]. The curve depicts the relationship between life expectancy and income and shows that the relationship is non-linear with life expectancy in wealthier countries being less sensitive to variations in average income. Many analyses of IMR declines over the last three decades have used the logarithmic transform of IMR [2-7]. And many studies of infant mortality inequalities among sub-population within countries have done the same [3,8].

A moment's reflection reveals that an infant mortality rate cannot keep declining linearly forever, since at some point it would reach zero, and it can never be negative. Although the logarithmic transformation is convenient, there is no evidence that logarithmic transformations are the most appropriate transformations for infant mortality rate time trends.

Whether or not IMR time series require logarithmic transformations has important implications for analyses of convergence of IMR both across countries and among sub-populations within countries. If IMR decline is exponential and requires a logarithmic transform, this implies more rapid IMR convergence. In contrast, if IMR decline is closer to linear, IMR convergence is expected to be slower.

**Figure 1:**
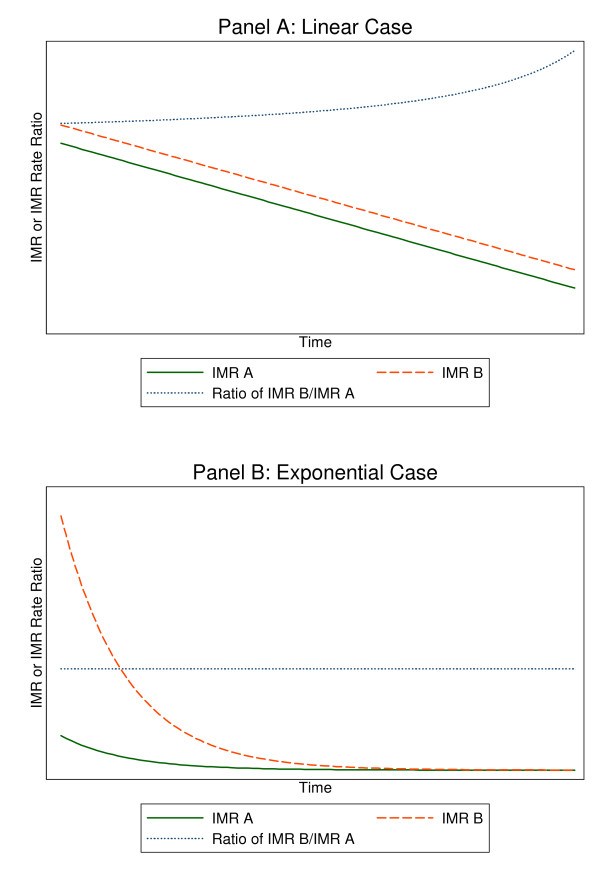
**Graphical depiction of how relative rate ratios evolve over time**. In panel A, two populations begin a linear process of IMR decline that maintains an equal gap and a constant decrement in IMR at each period. In Panel A, the rate ratio increases asymptotically as the lower rate ratio approaches zero. In panel B, the populations experience exponential decline in IMR with the same coefficient of decay but with population B starting from a higher infant mortality. In Panel B, the gap declines to nearly zero, but the rate ratio remains constant.

The shape of IMR declines also has implications for analyses of relative versus absolute gaps in IMR over time. Suppose IMR is declining in two populations called "A" and "B", and there is a gap so that B is higher than A. A rate ratio, B/A, is a measure of relative inequality, and a gap measure B-A is a measure of absolute inequality. There have been recent calls for researchers and policymakers to focus on relative gaps in health outcomes, including IMR, to assess health inequalities over time [9,10]. It is argued that absolute gaps in health outcomes such as IMR give a misleading impression of progress. Consider the extreme, and impossible, case that IMR declines are linear, as shown in Figure 1, Panel A. where population A obeys IMR_A _= A_0 _- *θ *× Time and population B obeys IMR_B _= B_0 _- *θ *× Time. Suppose there is a gap so that A_0_<B_0 _. In this case, population A will approach zero first, driving the denominator of IMR_A_/IMR_B _to zero and the quotient to infinity. Researchers describing rate ratios in this era would be able to perpetually document how the IMR rate ratio each year was larger than it ever was before. This appears to be happening even now [11]. If instead, the two populations have exponentially declining infant mortality rates, IMR_A _= exp(A_0 _- *θ *× Time) and IMR_B _= exp(B_0 _- *θ *× Time) and there is now a closing gap between population B and A so that A_0_<B_0_, the situation would be as shown in Figure 1, Panel B and the gap will narrow but the rate ratio will remain constant at exp(A_0_-B_0_) no matter how small the IMRs became. In this situation pessimists would be able to publish paper after paper decrying the lack of progress in relative rate ratios of IMR and optimists would be able to celebrate gaps that got smaller and smaller. In order for the IMR rate ratio to fall instead of remain constant it would be necessary for there to be a time-dependent shift in the slope *θ*, helping only the disadvantaged group so that |*θ*_A_|<|*θ*_B _|. Appendix 1 gives more mathematical details about the difference between absolute and relative measures of unequal trends in mortality.

In this paper, our objectives are twofold. First, we assess whether IMR decline as experienced in the 20^th ^century was a process of exponential decline in which case past analyses were wise to use logarithmic transformations of IMR in linear regressions. This objective will be met in the process of determining the best transformation to fit time trends in IMR reduction from 18 countries that span most of the 20^th ^century. Second, we examine how important it is to use the proper transformation of IMR trends in models. We do this by comparing regressions of 1n(IMR) on same year GDP per capita against appropriately transformed models. We demonstrate that the logarithmic transformation of IMR declines is seldom appropriate. And we show that assuming that IMR declines are logarithmic without checks can induce biases.

## Methods

We use two separate datasets for this analysis. First, we obtain IMR time series from the Human Mortality Database (HMD) . A collaborative project between the University of California, Berkeley and the Max Planck Institute for Demographic Research, the HMD provides detailed mortality time series for populations where death registration and census data are virtually complete. The methods used to compute the mortality rates and life tables in the HMD are described elsewhere [12]. We note only that in the absence of complete data, demographers frequently rely on interpolation to patch together annual mortality estimates from a collection of overlapping household surveys. An analysis of heavily interpolated data would yield limited insight on the best transformation to fit time trends in infant mortality reduction. The second dataset we use is the historical GDP per capita time series derived by Angus Maddison . The data are in 1990 International Geary-Khamis dollars. Details on the data and the sources for the time series are available elsewhere [13-17]. The selection of countries for this analysis is driven by data availability. All countries with IMR data and GDP per capita data for at least half of the 20^th ^century are included.

Box and Cox developed the following transformation:



arguing that the transformation on a dependent variable could make the residuals more closely normal and more homoskedastic [18]. Because the transformation embeds a number of popular functional forms including the reciprocal, logarithmic and square root transformations, it has also been used as a method for testing functional form [19]. When *λ *= 1, the Box-Cox transformation amounts to a simple linear model. As *λ *approaches -1, 0 and 1/2, the Box-Cox transformation converges to reciprocal, logarithmic, and square root transformations, respectively. (To see the limit of (a^*λ*^-1)/*λ *as *λ *→ 0, it is necessary to first Taylor expand both log(a) and BoxCox(a) and then observe their identity in the limit.) One disadvantage of the Box-Cox transformation is that it breaks down when zero or negative values must be transformed but this is not an issue in the analysis of IMR data.

We apply the Box-Cox transformation to IMR and then use maximum likelihood methods to identify the best fitting value of *λ *for the IMR time series for the 18 wealthy countries listed in Table 1 as well as for the pooled sample. We also use a likelihood ratio test to test the value of *λ *for each country and for the pooled sample against the null that *λ *= 0 (logarithmic model) and against the null that *λ *= 1 (linear model). In addition, to demonstrate the importance of selecting the proper transformation, for analyses of IMR decline, we run regressions of ln (IMR) on same year GDP per capita and compared the resulting coefficients with those resulting from regressions of Box-Cox transformations of IMR with optimal *λ *on same year GDP per capita.

**Table 1:** Results from Box-Cox models in which (IMR*^λ^*-1)/*λ *is regressed against time

**Country**	**Years**	*λ*	**Chi-squared for null that *λ *= 0** **(Logarithmic model)**	**Chi-squared for null that *λ *= 1** **(Linear Model)**
Whole sample		0.077	(22.050) ***	(2131.57) ***

Australia	1921–1999	0.249	(37.750) ***	(153.650) ***

Austria	1947–1999	0.102	(9.200) ***	(165.330) ***

Belgium	1900–1913 1918–1999	0.379	(67.180) ***	(130.450) ***

Canada	1921–1999	0.151	(50.290) ***	(288.830) ***

Denmark	1900–1999	0.260	(40.530) ***	(169.090) ***

Finland	1900–1999	0.356	(58.640) ***	(130.600) ***

France	1900–1999	0.354	(52.030) ***	(118.600) ***

Italy	1900–1999	0.587	(122.850) ***	(63.540) ***

Japan	1947–1999	-0.321	(62.000) ***	(238.210) ***

Netherlands	1900–1999	-0.092	(10.680) ***	(342.410) ***

New Zealand	1947–1999	0.591	(49.640) ***	(27.030) ***

Norway	1900–1999	0.364	(90.730) ***	(169.360) ***

Portugal	1940–1999	0.523	(74.340) ***	(64.170) ***

Spain	1908–1999	0.450	(71.570) ***	(82.540) ***

Sweden	1900–1999	0.192	(39.310) ***	(234.810) ***

Switzerland	1900–1999	0.133	(41.060) ***	(352.000) ***

UK	1900–1999	0.136	(34.220) ***	(310.440) ***

*USA*	1933–1999	0.040	(0.990)	(168.680) ***

No mortality data are available for Belgium from 1914–1918. USA has IMR time series that is best fit as logarithmic. ***p < 0.01 for likelihood ratio test for null that *λ *= 0 and *λ *= 1.

## Results

**Figure 2:**
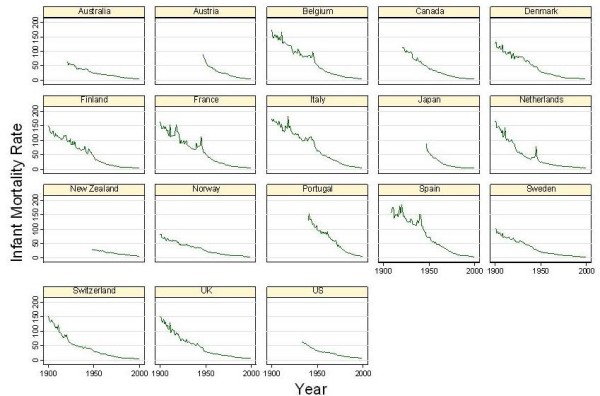
**20th Century Infant Mortality Decline: Graphs by Country**.

Figure 2 shows the 20^th ^century decline in IMR for the 18 countries included in this analysis. The length of the time series depends on the data available. While data for the entire 20^th ^century are available for Belgium, Denmark, Finland, France, Italy, Netherlands, Norway, Sweden, Switzerland, and the United Kingdom, shorter time series are available for Australia, Austria, Canada, Japan, New Zealand, Portugal, Spain, and the United States. From simple visual inspection of the IMR time series, it is difficult to discern any general rule of curvature in the pattern of decline over time. It is not possible to visually establish either the linearity or log-linearity of the IMR declines in these countries.

Table 1 shows the results of the Box-Cox models where (IMR*^λ^*- 1)/*λ *is regressed against year for each country. The third column in Table 1 includes the values of *λ *for each country and for the pooled sample that yield the most appropriate transformations. The fourth and fifth columns in Table 1 include the results of chi-squared tests comparing the best fit *λ *for each country and for the pooled sample for the null that *λ *= 0 (logarithmic model) and for the null that *λ *= 1 (linear model). Infant mortality rate decline during the 20^th ^century is best described as an exponential decline only for the United States. For the remaining 17 countries included in our study, the best transformations of IMR decline are neither linear nor logarithmic. The best fit for the pooled sample of IMR decline occurs with a transformation of (IMR^0.077^-1)/0.077.

The results highlighted in Table 1 show that logarithmic transformation of 20^th ^century IMR decline is appropriate for only one out of 18 countries. But this begs another question; does erroneously applying logarithmic transformation to IMR decline matter? Table 2 illustrates that biases can result from wrongly applying logarithmic transformation to IMR time series. Table 2 compares coefficients on GDP per capita based on two alternative assumptions about the curvature of IMR time series. Demographers have been studying the IMR versus GDP relationship ever since Preston [1,2,20], and nearly all studies use log-transformed IMR. In both models the independent variables are a constant, time in years, and GDP per capita in $1000 per person. In the Box-Cox models, the dependent variable is (IMR*λ*-1)/*λ *with*λ *equal to the best fitting value in the first model whereas Ln(IMR) is used in the second model. Table 2 highlights that the gaps in the coefficients on GDP per capita can be important. It reveals how far off one can be by naively assuming that Ln(IMR) is the best transformation. Note that in 2 countries, UK, and Australia, Box Cox models reveal that the coefficient on GDP per capita is actually positive instead of negative when controlling for secular time trends. That recessions are sometimes good for health is not a general phenomenon, but is increasingly recognized as occurring in high income countries for some health indicators [21,22]. This effect would have been masked by analysis that naively invoked a log transform for IMR.

**Table 2:** A comparison of coefficients on GDP per capita by IMR transformation

**Country**	**Years**	**Box-Cox** **IMR transformation** *λ *= **"Best Fit"**	**Log IMR transformation** *λ *= **0**	
		**Coefficient on GDP per capita**	**Coefficient on GDP per capita**	**Coefficient gap**

Whole sample		-0.065 ***	-0.078 **	**0.013**

Australia	1921–1999	0.217 ***	-0.034 **	**0.251**

Austria	1947–1999	0.151 ***	0.079	0.072

Belgium	1900–1913 1918–1999	-0.070 ***	-0.09 **	**0.02**

Canada	1921–1999	-0.038 ***	-0.051 **	**0.013**

Denmark	1900–1999	-0.057 **	-0.06 **	**0.003**

Finland	1900–1999	-0.088 ***	-0.106 **	**0.018**

France	1900–1999	-0.084 ***	-0.11 **	**0.026**

Italy	1900–1999	-0.151 ***	-0.132 **	**-0.019**

Japan	1947–1999	-0.020 ***	0.026	-0.046

Netherlands	1900–1999	-0.023 ***	-0.004	-0.019

New Zealand	1947–1999	0.035	0.068 *	-0.033

Norway	1900–1999	-0.004	-0.046 **	0.042

Portugal	1940–1999	-0.366 ***	-0.233 **	**-0.133**

Spain	1908–1999	-0.127 ***	-0.154 **	**0.027**

Sweden	1900–1999	-0.062 ***	-0.062 **	**0**

Switzerland	1900–1999	-0.009	-0.031 **	0.022

UK	1900–1999	0.145 ***	-0.031 **	**0.176**

*USA*	1933–1999	-0.009	-0.009	0

No mortality data are available for Belgium from 1914–1918. USA has IMR time series that is best fit as logarithmic. Bold coefficient gaps are for countries where coefficients are significant in both models.***p < 0.01, ** p < 0.05, *p < 0.10 for null that coefficient = 0.

## Discussion

For the countries and the years studied, the logarithmic transformation of IMR is seldom appropriate. Furthermore the results show that the logarithmic assumption can lead to biases in estimating the coefficient of IMR on covariates like GDP per capita. IMR decline in 18 countries during the 20^th ^century was for the most part neither linear nor an exponential decline. Only one of 18 IMR time series was best transformed as logarithmic. Although the logarithmic transformation of IMR is convenient and commonly used, we caution analysts against habitually taking logs. Statistical analyses of IMR time series should assess the robustness of findings to transformations other than the log transform and such assessments should be undertaken for all IMR time series separately.

This analysis is also relevant to the debate about what to expect when comparing IMR decline across multiple populations. As Figure 2 shows, historically, infant mortality decline occurred at a variable pace. The transition from IMR of 200 to IMR of less than 10 appears to have been the result of a heterogeneous process that was neither always linear nor always exponential throughout the 20^th ^century. A linear process would have meant that societies engaged in improving infant health found it possible to achieve the same size decrements each year regardless of whether IMR was low or high. An exponential process would imply that societies could generally lower IMR more when IMR was higher and less when it was lower. The history of IMR decline suggests that the timing of the process is heavily influenced by punctuated innovations in public health that disseminate laterally across countries [23,24]. Innovations in public health can provide permanent alterations in mortality levels and alterations in the pace of decline. By the same token, new epidemics such as the HIV epidemic can also punctuate the process.

The present state of the world reveals vast global disparities in infant mortality despite the wide availability of public health knowledge. Differences in material resources certainly provide a large part of the explanation for how international populations can share the same knowledge but achieve disparate mortality rates [4]. Differences in the efficiency of social institutions and health systems can also enable countries with similar resource levels to register disparate mortality levels [2]. What remains particularly perplexing is those intra-national disparities where populations share similar resource levels and health technology but achieve different health outcomes in various regions of the same country.

Tracking progress in health equity requires an objective measure of disparities that will accurately reflect progress when progress occurs. The analysis in this paper indicates that tracking the rate ratio in infant mortality rates (a relative measure) may fail to reflect progress. As shown in Figure 1, the rate ratio can increase asymptotically even though there is a constant gap and equal IMR decrements each year in the linear case or the rate ratio can remain constant even though the gap is narrowing and there are equal percentage drops in IMR every year in the exponential case. This study of the general pattern of IMR decline revealed that the average of 18 countries was for IMR to decline neither linearly nor exponentially. The best fit for data encompassing most of the twentieth century occurred overall when IMR^0.077 ^was linear in time. If there were one universal pattern of IMR decline, and it behaved like this best fit model, then the rate ratio of IMR rate ratios (higher divided by lower) would never decline. However there is substantial heterogeneity in the exponent on IMR across countries, and furthermore it is unlikely that the best fitting exponent for any single country remains constant across the span of a century. Assessing the degree of mortality convergence across populations in the 21^st ^century will require more sophisticated measures than rate ratios.

## Conclusion

This analysis warns of two hazards in the study of infant mortality rates. The first hazard is to assume that the best transformation of IMR for regression analysis is logarithmic. The second hazard is to assume that the rate ratio is a complete reflection of health disparities between populations. Our recommendations would be for future studies to proceed by always checking a Box-Cox transformation of IMR and using the best-fit Box-Cox parameter. Furthermore, an accurate reflection of disparities would need to supplement relative measures like rate ratios with absolute gap measures as well as measures of how the various aspects of the health system differentially affect the health of subpopulations.

## Competing interests

The authors declare that they have no competing interests.

## Authors' contributions

DB designed the study. Both authors contributed to data manipulation, data analysis, interpretation of findings and drafting of manuscript. Both authors read and approved the final manuscript.

## Appendix

The general formula for the derivative of a quotient f(t)/g(t)

Is given by: (f/g) [f'/f - g'/g].

Consider then the quotient of two declining linear functions: (B-bt)/(A-at)

The derivative for this quotient is:



Which is positive as long as B>bt and A > at and aB > bA indicating that the quotient will be increasing for the situation shown in Figure 1 where B > A and the slope a= b.

Now consider the ratio of two declining exponential functions: R= [e^(B-bt)^/e^(A-at)^]

The derivative of this quotient equals R [-b + a]. The sign of this derivative is positive if a> b. If a = b, the derivative is zero and the quotient never changes from its equilibrium value of e^(B-A)^.
